# A study on possible use of *Urtica dioica* (common nettle) plant as polonium ^210^Po and lead ^210^Pb contamination biomonitor in the area of phosphogypsum stockpile

**DOI:** 10.1007/s11356-015-5879-3

**Published:** 2015-12-09

**Authors:** Grzegorz Olszewski, Alicja Boryło, Bogdan Skwarzec

**Affiliations:** Laboratory of Environmental Analytics and Radiochemistry, Department of Environmental Chemistry and Radiochemistry, Faculty of Chemistry, University of Gdańsk, Wita Stwosza 63, 80-308 Gdańsk, Poland

**Keywords:** *Urtica dioica*, Soil, ^210^Po, ^210^Pb, ^210^Po^/210^Pb, Bioconcentration, Phosphogypsum

## Abstract

The aim of this study was to test a possible use of *Urtica dioica* (common nettle) plant as a biomonitor of polonium ^210^Po and lead ^210^Pb contamination near phosphogypsum stacks by determining concentrations of these radionuclides in samples collected from the area of phosphogypsum stockpile in Wiślinka (northern Poland). The ^210^Po and ^210^Pb contents in roots depended on their concentrations in soils. Bioconcentration factor values from soil to root of the plant did not depend on ^210^Po and ^210^Pb contents in soils that leads to the conclusion that different polonium and lead species have different affinities to *U. dioica* plants. The main sources of both analyzed radionuclides in green parts of plants are wet and dry air deposition and transportation from soil. The values of ^210^Po/^210^Pb activity ratio indicate natural origin of these radioisotopes in analyzed plants. ^210^Po and ^210^Pb concentration in *U. dioica* roots is negatively weakly correlated with distance from phosphogypsum stockpile.

## Introduction

**Fig. 1:**
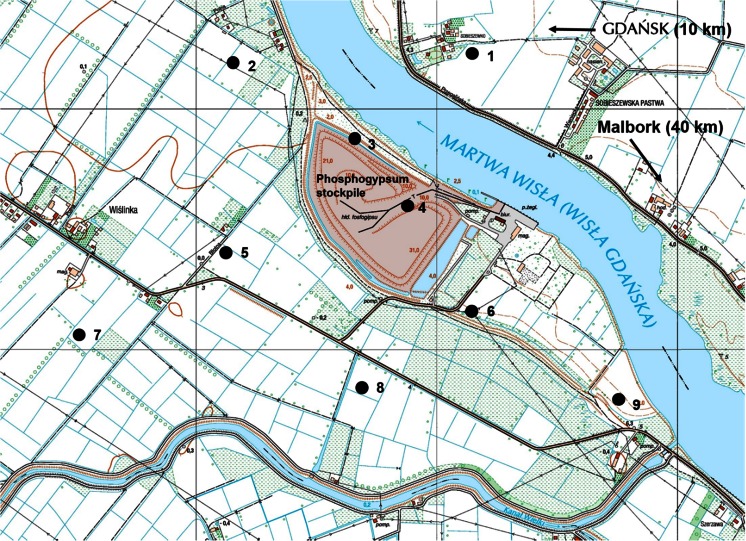
Sample collection sites

Phosphogypsum consists mainly of CaSO_4_·2H_2_O and is a by-product of phosphoric acid production from phosphate rocks (Hull and Burnett 1996). It is usually stored on stacks in specially designated areas. The phosphogypsum stockpile in Wiślinka (northern Poland) contains about 16 million tons of phosphogypsum (Boryło et al. 2013). It is located between the Martwa Wisła river and farm fields, close to the Gdańsk agglomeration (Fig. 1). Phosphate rocks are the starting material for the production of all phosphate products and the main source of phosphorus for fertilizers. They are characterized by high content of natural alpha radioactive elements, especially from ^238^U decay chain (^226^Ra, ^222^Rn, ^210^Po) and beta emitter ^210^Pb. The essence of radiotoxicity of the phosphogypsum is gamma radioactivity and high content of natural radioactive elements which could be leached by rain and bioaccumulated in plants, animals, and humans. In the process of phosphoric acid production, about 80 % of uranium is associated with the phosphoric acid fraction, while about 90 % of the ^210^Po and ^210^Pb is bound to the phosphogypsum fraction (Azouazi et al. 2001; Baxter 1996; Hull and Burnett 1996). Phoshphogypsum stockpile in Wiślinka is considered to be one of the main contaminators of the Martwa Wisła river. Our previous researches indicate that it might have serious radiological impact on the local environment. Phosphogypsum can be moved by the wind, and radionuclides might be leached by wet precipitation and transported through groundwaters to plants where they are accumulated (Bem 2005; Boryło et al. 2009, 2012, 2013; Skwarzec et al. 2010; Boryło and Skwarzec 2011; Olszewski et al. 2015).

Both ^210^Po and ^210^Pb are natural radionuclides, daughters of ^238^U decay series. Their half-lives are 138.38 days for ^210^Po and 22.3 years for ^210^Pb (Boryło et al. 2013). These natural radionuclides are found in varying concentrations in soil, sand, sediment, and natural water and constitute an important component of the natural background radiation. They are known to significantly contribute to the radiation dose of the population (Rajashekara and Narayana 2010). The main source of ^210^Po and ^210^Pb in the atmosphere is ^222^Rn emanation from the ground. ^210^Po and ^210^Pb return to the earth as dry fallout or are washed out in the rain. Important anthropogenic sources of these radionuclides are burning of fossil fuels, tetraethyl lead in petrol, dust storms, refineries, superphosphate fertilizers, the sintering of ores in steelworks, and the burning of coal in coal-powered power stations (Boryło et al. 2012). ^210^Po is highly toxic, and its presence in soils may be traced to the decay of radionuclides of the ^238^U chain in the soil (Aslani et al. 2005). Lead is widely distributed in the earth’s crust, but the main ore is galena, PbS, PbO, and PbO_2_. Carbonates, chlorophosphates, sulfates, sulfatocarbonates, and uosilicates are all less abundant than galena (Jia and Torri 2007). The possible different chemical behavior of both ^210^Po and ^210^Pb in the water column is characterized by a stronger affinity of ^210^Po for particles than its precursor, ^210^Pb (Gasco et al. 2002). The usual atmospheric input by rain has ^210^Po/^210^Pb activity ratio of 0.1–0.2 (Jia et al. 2003). The value of activity ratio higher than 1.0 must be affiliated with biogeochemical processes that can control the distribution of the two radionuclides (Jia et al. 2003).

The main aim of this work was to establish a possible use of *Urtica dioica* (common nettle) plants as ^210^Po and ^210^Pb contamination bioindicator in the area of phosphogypsum stockpile and to examine the impact of phosphogypsum stockpile on the surrounding environment. Additionally, the values of the ^210^Po^/210^Pb activity ratio and bioconcentration factor (BCF) and translocation factor (TF) were calculated in order to define both possible ^210^Po and ^210^Pb sources and level of their accumulation in plants.

## Materials and methods

### Sample collection and analysis

The *U. dioica* plant samples along with corresponding soils were collected from multiple locations in the area of phosphogypsum stack in Wiślinka (northern Poland) (Fig. 1) in September and October 2014. Over ground shoots of *U. dioica* plant die during winter, and it spends the winter in the form of underground rhizomes. In this case, we decided to collect samples in autumn when shoots are old and long enough exposed to air deposition. All of the collected samples had similar height (about 1.5 m). Control samples were collected in Malbork in Pomeranian Voivodeship, Poland. *U. dioica* was chosen for this research due to its commonness in Polish environment. Collected plants were divided into green parts and roots. Roots were washed with double deionized water in order to remove soil particles. Green parts were not washed due to examination of possible dry deposition impact. Only soil particles were removed. Before analysis, each plant sample was air-dried, homogenized using mortar, and dried in 60 °C. Soil samples were homogenized and passed through 0.25-mm sieve. From homogenized sample, three subsamples were weighted and enriched with approximately 20 mBq of ^209^Po yield tracer. Samples were mineralized using HCl, HNO_3_, and H_2_O_2_ mixture. Polonium was electrodeposited on silver discs according to the procedure presented by Skwarzec (1997, 2009). ^210^Pb was analyzed indirectly through its daughter’s ^210^Po activity measurement after 6-month storage. After this time, polonium was again electrodeposited on silver disc and ^210^Pb activity was calculated through ^210^Po activity (Skwarzec 1997, 2009).

### Measurement technique

The activities of ^210^Po were measured using an alpha spectrometer (Alpha Analyst S470) equipped in a surface barrier PIPS detector with an active surface of 300–450 mm^2^ placed in a vacuum chamber connected to a 1024 multichannel analyzer (Canberra—Packard, USA). Detector yield ranged from 0.30 to 0.40. In most of the used detectors, the resolution was 17–18 keV. Minimum detectable activity (MDA) for ^210^Po and ^210^Pb were 0.05 and 0.06 mBq g^−1^. The accuracy and precision of the radiochemical method were estimated to be less than 7 % by participation in international intercomparison exercises and analyses of IAEA materials. The precision between subsamples was estimated to be less than 3 % for all analyzed radioisotopes. ^210^Po activities were corrected for decay between deposition on silver discs and counting on alpha spectrometer. ^210^Po and ^210^Pb activities were calculated for sampling date. The value of BCF and TF were calculated as (Boryło et al. 2012)

1 $$ BCF=\frac{concentratio{n}_{root}}{concentratio{n}_{soil}} $$

2 $$ T{F}_{\frac{green\; part}{soil}}=\frac{concentratio{n}_{green\; part}}{concentratio{n}_{soil}} $$

3 $$ BC{F}_{\frac{plant}{soil}}=\frac{concentratio{n}_{plant}}{concentratio{n}_{soil}} $$

4 $$ TF=\frac{concentratio{n}_{green\; part}}{concentratio{n}_{root}} $$

## Results and discussion

### ^210^Po and ^210^Pb in *U. dioica* roots, shoots, and soils

The obtained concentrations of ^210^Po and ^210^Pb in analyzed plants and soils samples are presented in Table 1. The values of ^210^Po concentrations ranged from 5.67 ± 0.11 to 34.81 ± 0.25 Bq kg^−1^ dry wt. in green parts, from 1.21 ± 0.05 to 69.09 ± 1.93 Bq kg^−1^ dry wt. in roots, and from 18.42 ± 0.35 to 258.34 ± 4.18 Bq kg^−1^ dry wt. in corresponding soils. ^210^Pb concentrations in analyzed *U. dioica* green parts, roots, and soils ranged from 10.73 ± 0.10 to 40.93 ± 0.41 Bq kg^−1^ dry wt., from 1.20 ± 0.15 to 57.54 ± 1.56 Bq kg^−1^ dry wt., and from 18.28 ± 0.45 to 273.55 ± 4.12 Bq kg^−1^ dry wt., respectively. For analyzed common nettle control sample collected in Malbork, the obtained ^210^Po results were 9.61 ± 0.18 Bq kg^−1^ dry wt. for green part, 3.09 ± 0.15 Bq kg^−1^ dry wt. for root, and 21.09 ± 0.63 Bq kg^−1^ dry wt. for soil while ^210^Pb activities were 16.24 ± 0.11, 2.73 ± 0.09, and 21.36 ± 0.22 Bq kg^−1^ dry wt., respectively.

**Table 1:** ^210^Po and ^210^Pb contents in analyzed *Urtica dioica* plants and soils (given with expanded standard uncertainty calculated for 95 % CI; *n* = 3)

Collection site	^210^Po [Bq kg^−1^ dry wt.]			^210^Pb [Bq kg^−1^ dry wt.]		
	Green part	Root	Soil	Green part	Root	Soil
1	33.72 ± 0.17	2.14 ± 0.11	21.45 ± 0.45	41.43 ± 0.23	1.52 ± 0.15	19.73 ± 0.21
2	17.11 ± 0.15	6.06 ± 0.15	18.52 ± 0.35	19.58 ± 0.13	5.43 ± 0.17	18.28 ± 0.45
3	6.19 ± 0.17	69.09 ± 1.93	258.34 ± 4.18	10.73 ± 0.10	57.24 ± 1.56	273.55 ± 4.12
4	5.67 ± 0.11	12.40 ± 0.54	105.37 ± 2.75	11.25 ± 0.11	9.15 ± 0.23	110.75 ± 2.13
5	34.81 ± 0.25	1.21 ± 0.05	18.42 ± 0.35	40.93 ± 0.41	1.20 ± 0.15	19.11 ± 0.26
6	26.94 ± 0.21	5.15 ± 0.36	70.69 ± 2.43	31.05 ± 0.21	3.99 ± 0.10	77.73 ± 1.45
7	29.28 ± 0.22	2.32 ± 0.17	19.11 ± 0.54	38.30 ± 0.21	2.01 ± 0.35	17.62 ± 0.11
8	19.36 ± 0.25	5.18 ± 0.19	50.92 ± 1.76	21.37 ± 0.16	4.34 ± 0.14	51.63 ± 0.65
9	22.40 ± 0.28	2.72 ± 0.10	24.90 ± 0.54	29.23 ± 0.18	2.65 ± 0.24	25.12 ± 0.31
Control sample	9.61 ± 0.18	3.09 ± 0.15	21.09 ± 0.63	16.24 ± 0.11	2.73 ± 0.09	21.36 ± 0.22

### Comparison with other studies on ^210^Po and ^210^Pb uptake by plants

In Huleva, Spain, ^210^Po activities in *Spartina densiflora* plants were surveyed and obtained results ranged from 5.58 ± 0.41 to 45.2 ± 4.5 Bq kg^−1^ dry wt. in whole plant (Martinez-Aguirre et al. 1997). In 2011, samples of different plants (meadow, hygrophilous, edible, ruderal plants, and corn) were collected around phosphogypsum stack in Wiślinka and surveyed on polonium concentrations. Higher activities of ^210^Po were measured in roots of analyzed plants (from 6.4 ± 0.3 to 89 ± 1 Bq kg^−1^ wet wt. for roots and from 2.1 ± 0.1 to 51 ± 1 Bq kg^−1^ wet wt. for green parts). The only exception were edible plants where higher ^210^Po contents were measured in green parts than in roots (2.2 ± 0.1 and 1.2 ± 0.1 Bq kg^−1^ wet wt., respectively). The highest ^210^Po concentrations were noticed in ruderal plants collected from sewage sludge that covers the phosphogypsum stockpile (Boryło et al. 2012). In Finland, ^210^Po and ^210^Pb activities in wild berries were measured and the highest activities were noticed in stems of the analyzed samples (from 30 to 60 Bq kg^−1^ dry wt., for ^210^Pb, and from 30 to 90 Bq kg^−1^ dry wt. for ^210^Po), the lowest in fruits (<10 Bq kg^−1^ wet wt. for both analyzed radionuclides), while in roots, the measured concentrations were between 50 and 100 Bq kg^−1^ wet wt. for ^210^Po and between 35 and 45 Bq kg^−1^ wet wt. for ^210^Pb (Vaaramaa et al. 2010). Both ^210^Po and ^210^Pb concentrations in edible plants vary depending on the type of plants (e.g., leafy, rooty) (Ekdal et al. 2006).

### The values of ^210^Po/^210^Pb activity ratios in *U. dioica*

The values of ^210^Po/^210^Pb activity ratio in analyzed common nettle *U. dioica* plants and soils are presented in Table 2. The values ranged from 0.50 ± 0.17 to 0.90 ± 0.08 in green parts, from 1.03 ± 0.13 to 1.53 ± 0.10 in roots, and from 0.91 ± 0.11 to 1.09 ± 0.09 in soils. In green parts, roots, and soils of control samples collected in Malbork, the calculated values of ^210^Po/^210^Pb activity ratio were 0.60 ± 0.09, 1.13 ± 0.11, and 1.00 ± 0.04, respectively, while in wild berries, roots and green parts collected in Finland were higher than 1. This is probably connected with the fact that ^210^Po is more available to plants roots than ^210^Pb. The authors suggest further research in this matter (Vaaramaa et al. 2009). The ^210^Po excess in green parts of Finnish wild berries suggests another source of this radioisotope than wet and dry deposition, probably connected with transfer from soil. Similar results were observed in *U. dioica* roots in this study. In all analyzed common nettle samples, the values of ^210^Po/^210^Pb activity ratio were higher than 1 that suggests that ^210^Po is more mobile in soil where the values of these ratios are close to 1 (Table 2). In green parts of *U. dioica* plants, values of ^210^Po/^210^Pb activity ratio were lower than 1 suggesting that wet and dry deposition is significant source of ^210^Po and ^210^Pb radioisotopes in common nettle stems and leaves. Values that are close to unity could suggest either higher rate of metal transfer from soils or impact of phosphogypsum stockpile. The value of ^210^Po/^210^Pb activity ratio in air deposition ranges from 0.03 to 0.05 (Vaaramaa et al. 2010). It is assumed that up to 80 % of natural ^210^Po and ^210^Pb radioactivity in wild plants is connected with wet and dry deposition of ^222^Rn decay products (Persson and Holm 2011). In analyzed *U. dioica* plants, we received very high Spearman’s correlation factors between ^210^Po and ^210^Pb activities: *r*_s_ = 0.97 in green parts, *r*_s_ = 1.00 in roots, and *r*_s_ = 1.00 in corresponding soils that confirm natural origin of these radionuclides. Spearman’s rank correlation is a non-parametrical alternative for Pearson’s correlation. It can be used to calculate the correlation between two variables that do not have normal distribution and are not linear. Moreover, Spearman’s rank correlation is resistant for outlier results (Corder and Foreman 2014).

**Table 2:** The values of ^210^Po/^210^Pb activity ratios in analyzed *Urtica dioica* plants (given with expanded standard uncertainty calculated for 95 % CI; *n* = 3)

Collection site	Value of ^210^Po/^210^Pb activity ratio		
	Green part	Root	Soil
1	0.81 ± 0.11	1.38 ± 0.09	1.09 ± 0.09
2	0.85 ± 0.08	1.12 ± 0.10	1.01 ± 0.04
3	0.58 ± 0.12	1.21 ± 0.06	0.94 ± 0.05
4	0.50 ± 0.17	1.35 ± 0.11	0.97 ± 0.04
5	0.85 ± 0.05	1.03 ± 0.13	0.96 ± 0.05
6	0.89 ± 0.06	1.29 ± 0.08	0.91 ± 0.11
7	0.75 ± 0.09	1.13 ± 0.15	1.08 ± 0.12
8	0.90 ± 0.08	1.15 ± 0.05	0.99 ± 0.07
9	0.80 ± 0.12	1.53 ± 0.10	1.01 ± 0.08
Control sample	0.60 ± 0.09	1.13 ± 0.11	1.00 ± 0.04

### The values of ^210^Po and ^210^Pb BCF and TF in *U. dioica*

TF, TF_green part/soil_ and BCF, BCF_plant/soil_ common nettle samples were calculated according to Eqs. (1) to (4). The values of obtained factors are presented in Tables 3 and 4. For ^210^Po, they ranged from 0.066 ± 0.003 to 0.327 ± 0.020 for BCF, from 0.024 ± 0.001 to 1.889 ± 0.036 for TF_green part/soil_, from 0.17 ± 0.01 to 1.96 ± 0.09 for BCF_plant/soil_, and from 0.090 ± 0.003 to 28.67 ± 1.18 for TF. In control sample, calculated TF and BCF values were 0.147 ± 0.006, 0.456 ± 0.015, 0.60 ± 0.03, and 3.11 ± 0.11, respectively. In case of ^210^Pb, the obtained values ranged from 0.063 ± 0.008 to 0.297 ± 0.010 for BCF, from 0.039 ± 0.001 to 2.174 ± 0.021 for TF_green part/soil_, from 0.184 ± 0.006 to 2.288 ± 0.401 for BCF_plant/soil_, and from 0.19 ± 0.01 to 34.10 ± 4.28 for TF. For control samples, these values were 0.128 ± 0.004, 0.760 ± 0.009, 0.888 ± 0.031, and 5.95 ± 0.20, respectively.

**Table 3:** BCF and TF values for ^210^Po in analyzed *Urtica dioica* plants (given with combined standard uncertainty)

Collection site	TF	BCF	TF_green part/soil_	BCF_plant/soil_
1	15.79 ± 0.82	0.100 ± 0.006	1.572 ± 0.034	1.67 ± 0.09
2	2.82 ± 0.17	0.327 ± 0.020	0.924 ± 0.019	1.25 ± 0.08
3	0.090 ± 0.003	0.267 ± 0.007	0.024 ± 0.001	0.29 ± 0.01
4	0.46 ± 0.03	0.118 ± 0.006	0.054 ± 0.003	0.17 ± 0.01
5	28.67 ± 1.18	0.066 ± 0.003	1.889 ± 0.036	1.96 ± 0.09
6	5.23 ± 0.37	0.073 ± 0.006	0.381 ± 0.013	0.45 ± 0.04
7	12.63 ± 0.91	0.121 ± 0.009	1.532 ± 0.044	1.65 ± 0.13
8	3.73 ± 0.14	0.102 ± 0.005	0.380 ± 0.014	0.48 ± 0.03
9	8.23 ± 0.47	0.109 ± 0.006	0.899 ± 0.203	1.01 ± 0.06
Control sample	3.11 ± 0.11	0.147 ± 0.006	0.456 ± 0.015	0.60 ± 0.03

**Table 4:** BCF and TF values for ^210^Pb in analyzed *Urtica dioica* plants (given with combined standard uncertainty)

Collection site	TF	BCF	TF_green part/soil_	BCF_plant/soil_
1	27.21 ± 2.63	0.077 ± 0.007	2.100 ± 0.025	2.177 ± 0.212
2	3.61 ± 0.12	0.297 ± 0.010	1.071 ± 0.011	1.368 ± 0.045
3	0.19 ± 0.01	0.209 ± 0.007	0.039 ± 0.001	0.248 ± 0.008
4	1.23 ± 0.03	0.083 ± 0.003	0.102 ± 0.002	0.184 ± 0.006
5	34.10 ± 4.28	0.063 ± 0.008	2.142 ± 0.036	2.204 ± 0.278
6	7.78 ± 0.20	0.051 ± 0.002	0.399 ± 0.008	0.451 ± 0.014
7	19.04 ± 3.33	0.114 ± 0.020	2.174 ± 0.021	2.288 ± 0.401
8	4.92 ± 0.16	0.084 ± 0.003	0.414 ± 0.006	0.498 ± 0.018
9	11.03 ± 1.00	0.105 ± 0.010	1.164 ± 0.016	1.269 ± 0.116
Control sample	5.95 ± 0.20	0.128 ± 0.004	0.760 ± 0.009	0.888 ± 0.031

#### Comparison with other ^210^Po and ^210^Pb TF and BCF studies

Al-Masri et al. (2008) published TF values between fruits and leaves of vegetables and soils for both ^210^Po and ^210^Pb. Due to atmospheric deposition, TF to fruits (from 0.024 to 0.14 for ^210^Pb and from 0.028 to 0.2 for ^210^Po) were smaller than to leaves (from 0.37 to 1.4 for ^210^Pb and from 0.3 to 1.0 for ^210^Po). The values of ^210^Po TF_green part/soil_ for *Menthea* L. and *Petroselinum crispum* were 0.20 ± 0.08 and 0.46 ± 0.20, respectively (Al-Masri et al. 2010). These values are similar to some of our previous results as calculated TF values for ^210^Po concentrations in plants from Wiślinka were in the range from 0.06 for edible plants to 1.36 for hygrophilous plants, while BCF values ranged from 0.22 for hygrophilous plants to 2.30 for edible plants (Boryło et al. 2012). Manigandan and Manikandan (2008) analyzed different wild plants and calculated BCF_plant/soil_ values for ^210^Po between 0.292 and 0.336.

#### Characteristics affecting ^210^Po and ^210^Pb bioavailability

Uptake of Po and Pb by plants can occur both through the root system and from atmospheric deposition through activity interception by external plant surfaces (Vandenhove et al. 2009). Large number of factors is known to control metal bioavailability in soils and their accumulation level in plants. These are soil and climatic conditions, plant genotype, and agronomic management, including active/passive transfer processes, sequestration and speciation, redox states, the type of plant root system, and the response of plants to elements in relation to seasonal cycles (Kabata-Pendias and Pendias 1984; Malik et al. 2010). One of the major factors that contribute to extent of the metals taken up by the plants is also the structure and type of the soil. Also, such factors as clay particles, metal solubility controlled by pH, amount of metal cation exchange capacity, organic carbon content, and oxidation state of the system are important in metal availability (Malik et al. 2010). Narayana et al. (2006) reported higher ^210^Po and ^210^Pb sorption in soils with increasing organic matter content that was confirmed by high correlation factors (0.62 for ^210^Pb and 0.70 for ^210^Po). Vaaramaa et al. (2010) conducted research of soil cores divided into litter, organic, illuvial, and eluvial horizons and showed that ^210^Pb activity is correlated with organic matter content in soil. In all analyzed soil horizons, high ^210^Pb activity correlations between organic matter content were proved (the highest 0.85 for eluvium). No relevant correlations between organic matter content and ^210^Po concentrations in soils were reported. Similar results were obtained for relationships between Mn, Fe, Al, and Pb contents and ^210^Po and ^210^Pb activities in soils. Radiolead is strongly correlated with these metal concentrations, while no significant correlations were received for ^210^Po activities (Vaaramaa et al. 2010). Berger et al. (1965) indicated that organic soils contain on average three times more ^210^Po than mineral soils. These differences might be associated with higher ^210^Po sorption on clay and organic matter. In case of ^210^Po and ^210^Pb, the bioavailability is mainly dependent on their content in soils, plant morphology, and the level of wet and dry precipitation.^210^Po and ^210^Pb activity concentrations in root and tuber crops, cereals, and legumes, where the edible portion is protected by inedible plant parts, should not be affected by air deposition containing both ^210^Po and ^210^Pb (Vandenhove et al. 2009).

#### TF and BCF values’ variability explanation

**Fig. 2:**
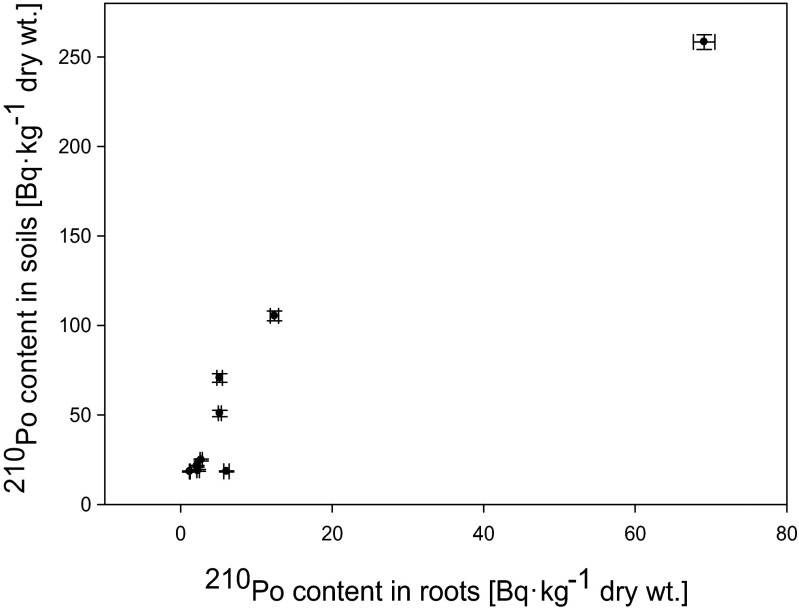
Relation between ^210^Po content in roots of analyzed *Urtica dioica* plants and soils (*r*_s_ = 0.72)

**Fig. 3:**
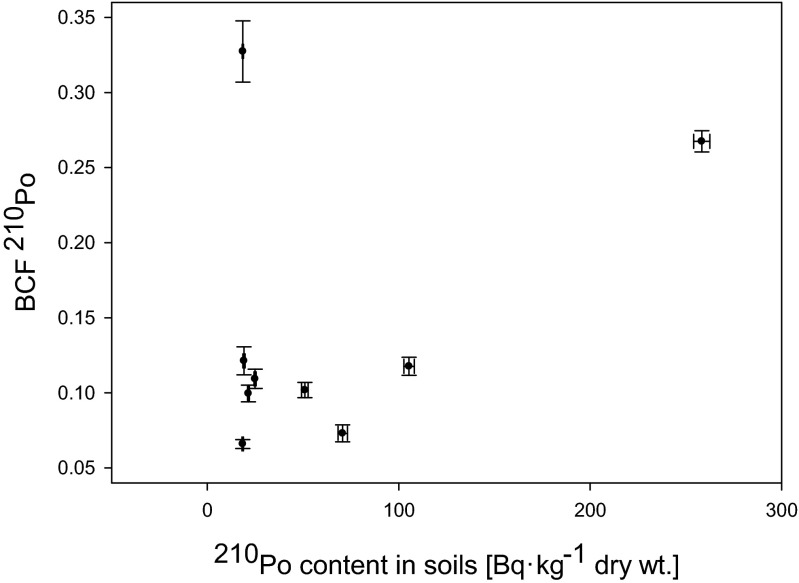
Relation between ^210^Po content in soils of analyzed *Urtica dioica* plants and BCF values (*r*_s_ = 0.17)

**Fig. 4:**
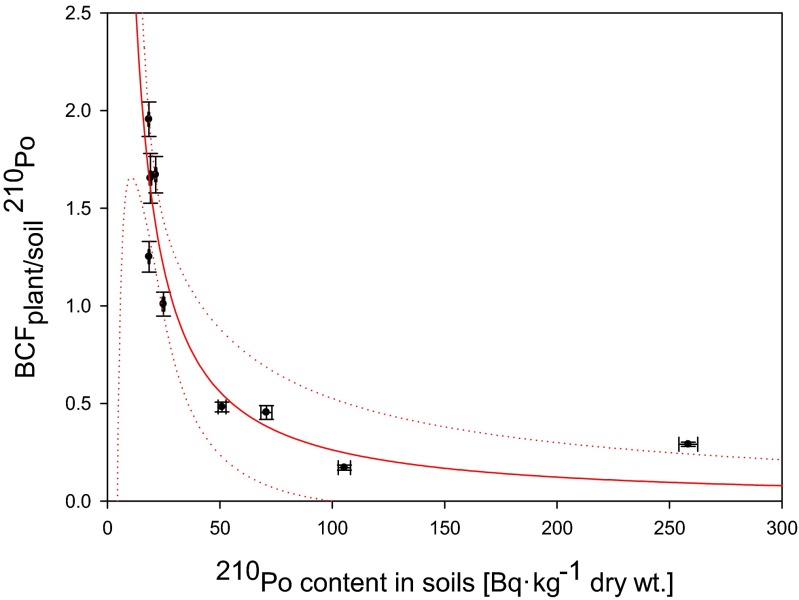
Relation between ^210^Po content in soils of analyzed *Urtica dioica* plants and BCF_plant/soil_ values (*y* = 4.044*x* ^−1.094^) (*r*_s_ = −0.92)

^210^Po and ^210^Pb concentrations in analyzed *U. dioica* samples are lower than their contents in corresponding soils. It is clearly seen that both ^210^Po and ^210^Pb activities in common nettle’s roots are dependent on their content in soils (*r*_s_ = 0.72 for ^210^Po and 0.65 for ^210^Pb) (Fig. 2), while the value of BCF does not exhibit this correlation (*r*_s_ = 0.17 for ^210^Po and 0.18 for ^210^Pb) (Fig. 3). BCF_plant/soil_ values are strongly and negatively correlated with ^210^Po and ^210^Pb concentrations in soils (*r*_s_ = −0.92 for ^210^Po and −0.93 for ^210^Pb) (Fig. 4). According to Chen et al. (2005). there are considerable differences in the uptake and translocation of long-lived radionuclides among different plant species. Perianez and Martinez-Aguirre (1997) reported that BCF_plant/soil_ factor in relation with ^210^Po contents in soil can be described using function

5 $$ {\mathrm{BCF}}_{\mathrm{plant}/\mathrm{soil}}=2.456{\left[{\mathrm{C}}_{\mathrm{soil}}\right]}^{-0.663} $$

This phenomenon is confirmed in our study on Fig. 4 where BCF_plant/soil_ factor is not linearly related with ^210^Po concentration in soil and can be described by function

6 $$ {\mathrm{BCF}}_{\mathrm{plant}/\mathrm{soil}}=4.044{\left[{\mathrm{C}}_{\mathrm{soil}}\right]}^{-1.094} $$

**Fig. 5:**
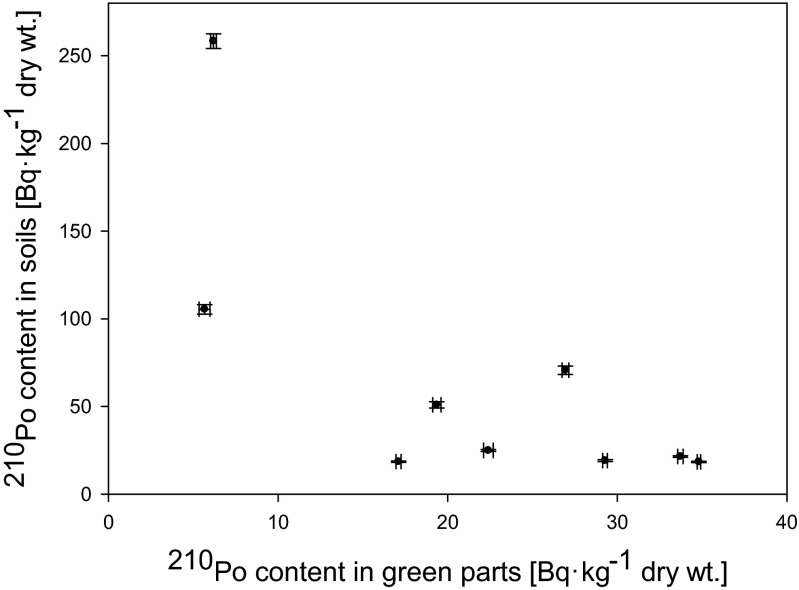
Relation between ^210^Po content in soils and green parts of analyzed *Urtica dioica* plants (*r*_s_ = −0.67)

**Fig. 6:**
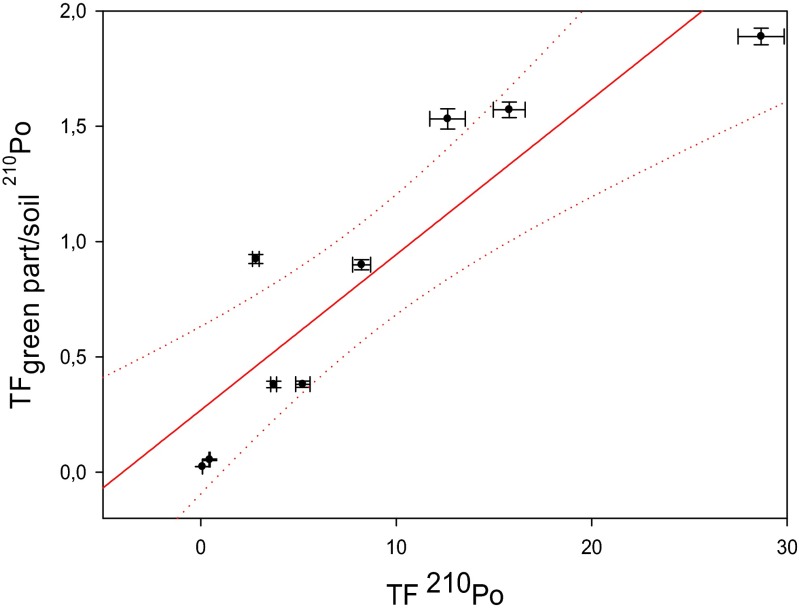
Relation between ^210^Po TF_green part/soil_ and TF values calculated for analyzed *Urtica dioica* plants (*r*_s_ = 0.90)

It is probably connected with the fact that plants readily uptake elements essential for their growth when substrate concentrations are low (Mengel and Kirkby 1979). Usually, plant uptake of non-essential elements is constant in this substrate concentration. Only in high substrate concentrations for both essential and non-essential elements it can decrease, leading to toxicity or death of a plant (Martinez-Aguirre et al. 1997). Even though, some non-essential elements can mimic an essential element resulting in non-linear relationship between soil concentration and BCF_plant/soil_ at low substrate concentrations (Sheppard and Sheppard 1985). This phenomenon in lesser extent can be also noticed in case of relation between BCF values and ^210^Po and ^210^Pb concentrations in soils (Fig. 3). Plants take up radionuclides of similar chemical forms as the essential nutrient and transport them to specific tissues based on the function of the element in plant metabolism that is reflected in its higher concentration in a particular part compared to others (Al-Kharouf et al. 2008). Another explanation could be connected with the level of wet and dry deposition on green parts of the plants and with differences in previously mentioned soil and plant characteristics that may affect bioavailability of metals (Persson and Holm 2011). According to Vandenhove et al. (2009). the soil type and texture are crucial for Pb BCF_plant/soil_ values, while in case of ^210^Po, this factor is highest on coarse-textured soils and lowest on fine-textured and organic soils. Our results suggest that *U. dioica* roots could be used as a biomonitor of ^210^Po and ^210^Pb contamination in exploratory studies even though there are differences in both radionuclide bioavailability from soils to roots. The highest BCF_plant/soil_ values were calculated for plants grown on soil with low both ^210^Po and ^210^Pb contents. Sample numbers 3 and 4 (Table 1) have significantly lower ^210^Po and ^210^Pb activities in green parts. Probable explanation is that the exposure to precipitation varies with the degree of covering of leaves. Roots serve as a natural barrier preventing the transport of many trace metals including radionuclides to the upper plant parts. The radionuclide translocation from roots to shoots is probably dependent on the species (Shtangeeva 2010). Relations between ^210^Po and ^210^Pb content in soils and in green parts of analyzed *U. dioica* plants is also negatively correlated (*r*_s_ = −0.67 for ^210^Po and −0.60 for ^210^Pb) (Fig. 5) that confirms differentiated impact of air deposition on these radioisotope contents in plant leaves. As a confirmation, we plotted TF and TF_green part/soil_ values. Received linear function and high *r*_s_ values (*r*_s_ = 0.90 for both radionuclides) suggest similar source of both ^210^Po and ^210^Pb in green parts of *U. dioica* (Fig. 6). According to these results, it is impossible to use common nettle’s leaves and stems as a biomonitor of possible phosphogypsum particle deposition from air as their impact is probably irrelevant when compared to air particles or metal transfer from soil. BCF and TF values received for control samples (Tables 3 and 4) indicate that soil characteristics and air deposition are mainly responsible for ^210^Po and ^210^Pb uptake.

### Impact of the phosphogypsum stack

**Fig. 7:**
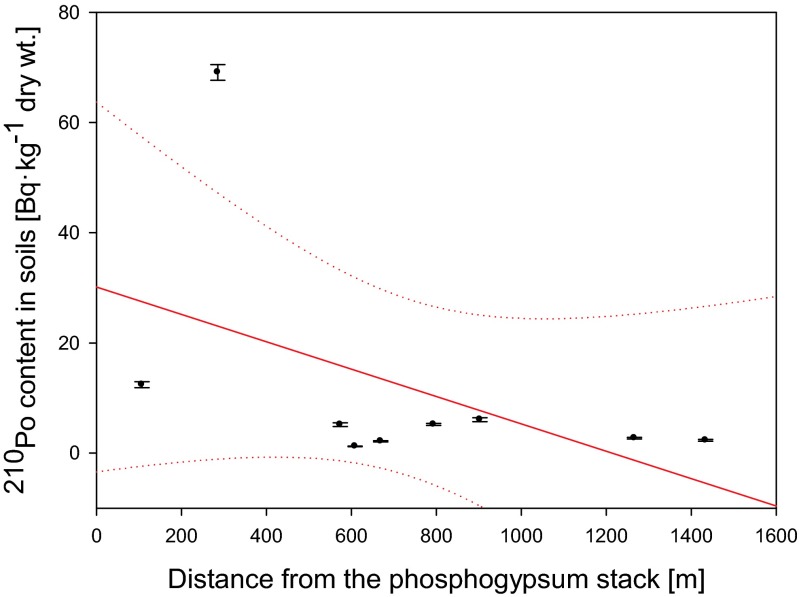
Relation between ^210^Po content in roots of analyzed *Urtica dioica* plants and distance from the phosphogypsum stockpile (*r*_s_ = −0.43)

**Fig. 8:**
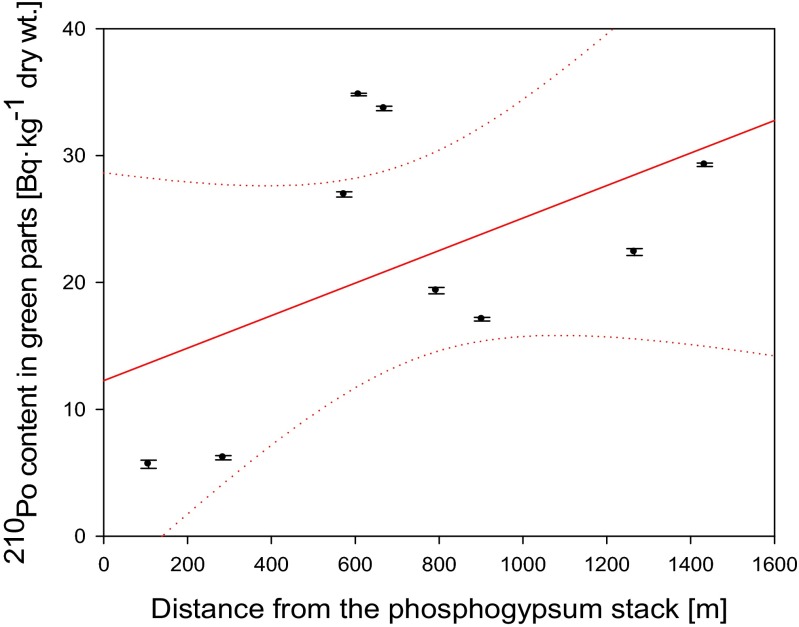
Relation between ^210^Po content in green parts of analyzed *Urtica dioica* plants and distance from the phosphogypsum stockpile (*r*_s_ = 0.37)

Possible impact of ^210^Po and ^210^Pb from phosphogypsum stockpile on green parts, roots, and whole plants of common nettle *U. dioica* in respect with distance from the stack was evaluated. The obtained results indicate that concentrations of both radionuclides in roots of analyzed plants are weakly but negatively correlated with distance from phosphogypsum stockpile (*r*_s_ = −0.43) (Fig. 7). Stronger correlations were obtained between ^210^Po and ^210^Pb concentrations in soils and distance from the stack (*r*_s_ = −0.61 and −0.78, respectively). In case of green parts of *U. dioica*, no relevant correlations were calculated (*r*_s_ = 0.37) (Fig. 8). Moreover, no significant correlations were observed for ^210^Po and ^210^Pb activities in whole plants (*r*_s_ = −0.20 and −0.08, respectively) that suggests the crucial impact of air deposition on ^210^Po and ^210^Pb activities in *U. dioica* plants.

## Conclusions

Polonium ^210^Po and lead ^210^Pb concentrations in analyzed plants and soils allow us to conclude that *U. dioica* roots can be used as a biomonitor of both ^210^Po and ^210^Pb soil contamination especially during exploratory surveys. We noticed that both radionuclides activities in roots were related to their concentrations in soils although BCF values did not present similar dependence. The relation between ^210^Po and ^210^Pb soil concentrations and BCF_plant/soil_ values is non-linear what confirms that some forms of Po and Pb can mimic essential elements for plant growth, and depending on soil characteristics, they can be more easily absorbed by plants. Another explanation could be connected with the level of wet and dry deposition on green parts of the plants. These facts prevent using common nettle’s leaves and stems as a biomonitor of possible phosphogypsum particles deposition. The decrease of ^210^Po and ^210^Pb concentrations in common nettle’s root was noticed with increasing distance from phosphogypsum stockpile. This relation was not confirmed for analyzed green parts that is probably connected with the impact of the air deposition. The problem of phosphogypsum stockpile is limited to the zone of maximum 400 m. The highest contents of both ^210^Po and ^210^Pb were measured in samples collected from the slopes of the stack. The values of ^210^Po/^210^Pb activity ratio confirm natural sources of these radionuclides in *U. dioica* plants.
